# Shedding of Infectious Borna Disease Virus-1 in Living Bicolored White-Toothed Shrews

**DOI:** 10.1371/journal.pone.0137018

**Published:** 2015-08-27

**Authors:** Daniel Nobach, Manon Bourg, Sibylle Herzog, Hildburg Lange-Herbst, Jorge A. Encarnação, Markus Eickmann, Christiane Herden

**Affiliations:** 1 Institute of Veterinary Pathology, Justus-Liebig-University, Giessen, Germany; 2 Institute of Virology, Justus-Liebig-University, Giessen, Germany; 3 Mammalian Ecology Group, Department of Animal Ecology and Systematics, Justus-Liebig-University, Giessen, Germany; 4 Institute of Virology, Philipps-University, Marburg, Germany; Division of Clinical Research, UNITED STATES

## Abstract

**Background:**

Many RNA viruses arise from animal reservoirs, namely bats, rodents and insectivores but mechanisms of virus maintenance and transmission still need to be addressed. The bicolored white-toothed shrew (*Crocidura leucodon*) has recently been identified as reservoir of the neurotropic Borna disease virus 1 (BoDV-1).

**Principal Findings:**

Six out of eleven wild living bicoloured white-toothed shrews were trapped and revealed to be naturally infected with BoDV-1. All shrews were monitored in captivity in a long-term study over a time period up to 600 days that differed between the individual shrews. Interestingly, all six animals showed an asymptomatic course of infection despite virus shedding via various routes indicating a highly adapted host-pathogen interaction. Infectious virus and viral RNA were demonstrated in saliva, urine, skin swabs, lacrimal fluid and faeces, both during the first 8 weeks of the investigation period and for long time shedding after more than 250 days in captivity.

**Conclusions:**

The various ways of shedding ensure successful virus maintenance in the reservoir population but also transmission to accidental hosts such as horses and sheep. Naturally BoDV-1-infected living shrews serve as excellent tool to unravel host and pathogen factors responsible for persistent viral co-existence in reservoir species while maintaining their physiological integrity despite high viral load in many organ systems.

## Introduction

Most of emerging viruses that are continuously detected belong to the RNA viruses and are often zoonotic in nature with epidemic or epizootic potential in case of transmission to livestock or humans [1–3]. Interestingly, approximately 50% of the highly pathogenic diseases caused by these agents affect the central nervous system [4–6]. Emerging viruses and also viruses highly pathogenic for animal species often arise from animal reservoirs, namely bats, rodents and insectivores. Thus, reliable animal models for the in vivo analysis of host-pathogen interactions in respective reservoir species and the mechanisms that drive crossing of species barriers are urgently needed. This could also allow characterization of transmission routes and maintenance in reservoir populations of these viruses. The order *Mononegavirales* comprises non segmented negative stranded RNA viruses with a considerable number of highly pathogenic viruses which reside inconspicuously in natural reservoirs, e.g. lyssaviruses, paramyxoviruses and henipaviruses in bats. In case of transmission to susceptible animals or humans they cause fatal disease [7, 8]. Borna disease virus-1 (BoDV-1) also belongs to the order *Mononegavirales* and was classified within an own and currently growing family named *Bornaviridae*. A new classification of this family with subdivision into 5 species has been proposed with the classical Borna disease virus-1 as part of the species *Mammalian 1 bornavirus* [9]. Recently, a variegated squirrel-derived bornavirus (VSBV-1) was found in association with the death of three people indicating the zoonotic potential for this newly discovered bornavirus [10]. Comparably to other reservoir-bound viruses of the order *Mononegavirales*, BoDV-1 infection can lead to a lethal neurological disorder in accidental hosts such as horses and sheep due to a severe immune mediated non purulent meningoencephalitis [11]. The strictly endemic course of Borna disease with seasonal appearance in spring and early summer, the varying incidence between years with peaks every three to five years as well as the highly conserved viral genome pointed to a natural reservoir for BoDV-1 already for a long time [12]. However, many studies in wild rodents did not reveal any signs of BoDV-1 infection in these species [13]. First evidence of natural BoDV-1 infection in small mammals was provided by the detection of BoDV-1 antigen and RNA in bicolored white-toothed shrews (*Crocidura leucodon*) originating from an endemic area in Switzerland [14, 15]. This was substantiated by a study based on a geographic information system analysis which connects the prevalence of Borna disease and the distribution of *C*. *leucodon* [16]. Recently, similar occurrence of BoDV-1 infection in *C*. *leucodon* in endemic areas in Bavaria and in Saxony-Anhalt [17, 18] further underlines the role of this shrew species as BoDV-1 reservoir. Overlapping feature of all BoDV-1-infected shrews—regardless of their endemic origin—is the widespread virus distribution not only in the central nervous system (CNS) but also in peripheral organs capable of shedding virus in secretions and excretions [15, 17, 18]. Experimental BoDV-1 infection of neonatal immune incompetent rats leads to a quite comparable mode of virus distribution [19]. In these animals, persistent infection is achieved by immune tolerance. Obvious neurological signs are lacking but behavioural deficiencies have been noted. In contrast, adult Lewis rats exhibit a severe neurological biphasic disease due to a non purulent meningoencephalitis closely resembling the accidental host situation. Certain mice strains develop a fatal neurological disease only after intracerebral infection of newborns [20, 21]. Thus, outcome of experimental BoDV-1 infection in rodents such as mice and rats depend on the species and even the particular strain and, the age at time point of infection. The latter is most likely explainable by the status of the immune system. This leads to significant differences in virus-host interactions resulting in variable clinical outcome and fatality of disease, reaction pattern of the immune system, virus distribution and shedding.

Whether natural BoDV-1 infection of *C*. *leucodon* may fit to any of the known experimental courses or even run a different and so far unknown way of infection remains unknown. Thus, clinical outcome, routes of virus shedding including demonstration of infectivity was characterized in BoDV-1-infected *C*. *leucodon*. This contributes to understand not only BoDV-1 pathogenesis but also serve as in vivo model for the analysis of general mechanisms of viral co-existence of reservoir-bound neurotropic viruses in physiologically normal appearing hosts.

## Material and Methods

### Animals

To further characterize viral maintenance in reservoir species, bicolored white-toothed shrews were caught alive. Trapping was performed at two sites in the administrative district of Swabia (permission No. 55.1-8646-2/75), a known endemic area for BoDV-1 infections and clinical apparent disease (Borna disease) in horses. After trapping, shrews were transported separately and put in husbandry. The animals were kept isolated from each other in single cages. They were kept in adapted standard cages type 4 and with respect to the natural requirements they were fed with a mixture of chicken heart muscle, chicken liver and insects. After an adaption period of 4 weeks and the initial veterinary care the animals stayed in husbandry for breeding. With respect to the high vulnerability to stress of wild-born animals, a health monitoring was installed. Once a day a visual examination of body condition, haired skin and behaviour was carried out and food intake was measured. Once a week body mass was recorded. The animals were monitored for any sign of direct abnormal behaviour or any indirect evidence like altered food intake, altered skin care or loss of body weight. In case of suffering, an early humane endpoint scheme adopted from laboratory rodents could be applied including euthanasia by anaesthesia conducted with CO_2_ and decapitating. A postmortem examination scheme with evaluation of gross and histologic lesions could be performed to evaluate the cause of death.

For comparative analysis, tissues from three naturally BoDV-1-infected dead bicolored white-toothed shrews from pest control (#2001 and #5017, [17] and another animal #5072 from the same stable as #5017) were used.

### Methods

At trapping, infection status of the animals was unknown therefore high hygiene standards were applied to avoid accidental transmission. To detect naturally BoDV-1-infected animals, first samples of skin surface were taken directly on first day in husbandry and screened for the presence of BoDV-1 RNA as described below. Non-infected animals were sampled in the same way. In animals caught in 2013 (group 1: female #2, male #5, female #6), after an adaption phase of one month, samples of saliva, lacrimal fluid, skin surface, urine and excrements from the BoDV-1-infected shrews were taken weekly over a period of 4 weeks as necessary veterinary care. Initial veterinary care could be reduced in animals caught in 2014 (#9, #10, #12) and only an initial sampling was performed. As health monitoring, possibility of long lasting virus shedding after at least more than 250 days in the husbandry was investigated and infected shrews (group 2: female #2, male #9, male #10, male #12) were sampled again. Quantitative amplification of BoDV-1 RNA was carried out by real time RT-PCR as described elsewhere [22] by using commercially available kits for RNA extraction and real-time reverse transcription polymerase chain reaction (QIAsymphony RNA Kit, OneStep RT-PCR kit, Qiagen).

Qualitative isolation of infectious virus was performed on rabbit embryonic brain cells (REB cells) according to Herzog et al., 1980 [23]. Briefly, cells were incubated with diluted samples from shrews no #2, #5, #6 and virus replication in REB cells was visualized by indirect immunofluorescence test [24]. Viral RNA was extracted from REB cells persistently infected with the isolated BoDV-1 by using commercially available kits for RNA-extraction (RNeasy Mini Kit, Qiagen) and was sequenced according to previous protocols [25]. The nucleotide sequences were submitted to GenBank database.

Phylogenetic studies were performed as described elsewhere [25] using the Phylogeny Inference Program package, PHYLIP [26]. Representative sequences of all five regional BoDV-1 subclusters were obtained from GenBank (Group 1A: L27077, AY374524; Group 1B: AY374551, AY374550; Group 2: AY374521, AY374531; Group 3: AY374519, AY374534; Group 4: U04608, AY374522; Borna Disease Virus-2 AJ311524). Firstly, SEQBOOT program was used for testing stability of the trees by bootstrap resampling analysis of 100 replicates. Secondly, genetic distances between each pair of sequences were calculated based on the Kimura two-parameter model, transition/transversion ratio of 2, computed with the DNADIST program. Thirdly, using the neighbour-joining method of the NEIGHBOR program a phylogenetic tree was generated and printed out as a consensus tree by the CONSENSE program. Finally, the phylogenetic tree was displayed using SEAVIEW [27].

Immunohistochemistry (IHC) was carried out using the monoclonal anti-BoDV-1 nucleoprotein (BoDV-1-N) antibody Bo18 as described elsewhere [28, 29]. In-situ hybridization (ISH) to detect genomic RNA and respective mRNA sequences of the BoDV-1-N gene was performed additionally applying established protocols [29].

All statistical analyses were performed using Statistica 10 software package (StatSoft, Tulsa, Oklahoma, USA). Female shrew #3 was excluded from the statistical analyses of relative body mass trend as it was used for breeding during the observation period and could therefore show changes of body mass due to pregnancy. Relative body mass trend was calculated by ratio of body mass of an individual at time point x to the body mass at day 1 of husbandry. Normality of data was assessed using Kolmogorov-Smirnov and Lilliefors tests. We used a Kruskal-Wallis test to assess significant differences in weekly body mass trend between individuals. We used Mann-Whitney-U test to test for significant differences relative in body mass trend between non-infected and infected shrews within the same week in husbandry. The criterion to accept statistical significance was p < 0.05.

### Ethics Statement

Animal husbandry and health management were performed in accordance with the German law and were declared to the Animal Welfare Officer of the University, additional ethical waiver of an ethical Animal Care and Use Committee was not required. Prior to animals capture, capture protocol and gathering of animals were approved and permitted by the administrative district of Swabia (permission No. 55.1-8646-2/75) for establishing an insectivore animal model. Additional approval by an animal ethics committee for capture of wild animals was not required. Capture of wild animals was performed by skilled veterinarians according to the “Guidelines for the capture, handling and care of mammals as approved by the American Society of Mammalogists” of the Animal Care Use Committee [30]. Animals were kept in an animal facility of the Philipps-University in Marburg, animal housing was licenced (Az LRV FD 83.4.1-19c 20/21) by the administrative district of Marburg-Biedenkopf according to the law (Animal Welfare Act = “Tierschutzgesetz”, §11) and to the guidelines of the Veterinarian Association for animal welfare (= “Tierärztliche Vereinigung für Tierschutz e.V.”). Only non-invasive diagnostic sampling procedures during routine veterinary care were applied that did not need to be additionally approved by an animal ethics committee.

Rabbit embryonic brain cells were generated in the early 1990s by S. Herzog and frozen until usage. Generation of these primary cells was licenced (Gi 23-1/89) by the administrative district of Giessen.

## Results

**Fig 1:**
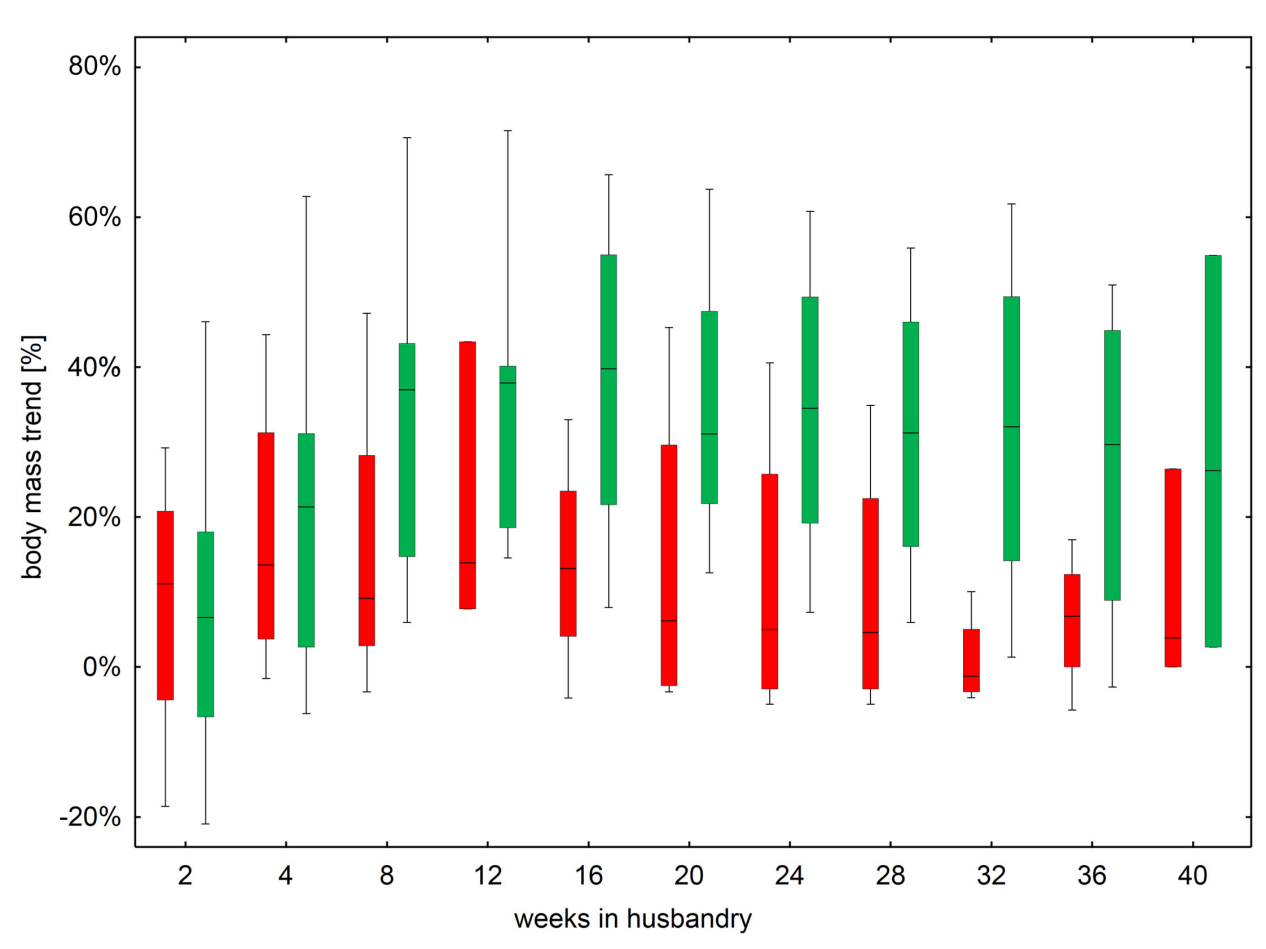
Relative body mass trend of non-infected and infected shrews. Relative body mass trend of non-infected and infected shrews at 11 time points (weeks in husbandry 2, 4, 8, 12, 16, 20, 24, 28, 32, 36, 40) with no differences in non-infected (demonstrated in green, Kruskal-Wallis-Test: H(10;42) = 4.3123; p = 0.9322) and infected animals (demonstrated in red, Kruskal-Wallis-Test: H(10;50) = 6.8237; p = 0.7420) and between groups (Mann-Whitney U-test, p > 0.06)

Eleven bicolored white-toothed shrews were caught (4 females [#2, #3, #6, #8], 7 males [#1, #5, #7, #9, #10, #12, #13]). An overview about the different shrews is given in S1 Table. As animals were integrated into husbandry at different time points, the observation period varied between the animals. In totally six out of eleven shrews (female #2, male #5, female #6, male #9, male #10, male #12) natural BoDV-1-infection was confirmed by detection of viral RNA, in three out of eleven shrews (female #2, male #5, female #6) additionally by detection of infectious virus. Two of the naturally infected shrews (male #5, female #6) died about 9 weeks after the start of the observation period (see below). The five other shrews did not exhibit any evidence for BoDV-1-infection, neither infectious virus nor viral RNA was detected at any time point investigated. During the whole observation period up to 600 days, activity and behaviour during day or night light regime and food intake did not differ between infected and non-infected animals. Furthermore, there was no significant difference of relative body mass trend between infected and non-infected individuals (Mann-Whitney U-test, p > 0.06 for each comparison) (Fig 1). There was also no significant difference of relative body mass trend between different weeks in husbandry of non-infected animals (Kruskal-Wallis-Test: H(10;42) = 4.3123; p = 0.9322) and between different weeks in husbandry of infected animals (Kruskal-Wallis-Test: H(10;50) = 6,8237; p = 0.7420). Body mass of the individual shrews are shown in S1 Fig. Six shrews (#2, #7, #9, #10, #12, #13), both infected and non-infected ones, developed focal alopecia after 4 to 5 months. Two animals of group 1 (#5, #6) were found dead without previous symptoms shortly after the initial health monitoring. Post mortem examination revealed intestinal invagination as cause of death in one case and hepatitis and pneumonia without known etiology in the other case.

Infected animals caught in 2013 received an intensive initial health monitoring including shedding of the virus for 4 weeks. In these three naturally infected shrews (#2, #5, #6), viral RNA was present in saliva, lacrimal fluid, skin swabs, urine and faeces as well as in the ground substrate from their lairs (Table 1). During the observation period viral RNA was consistently present in swabs from saliva and skin, however detection varied in urine, lacrimal fluid and was solely sporadically possible in faeces. Ct-values were lowest in samples of saliva.

**Table 1:** Detection of BoDV-1* RNA in naturally infected bicolored white-toothed shrews over a period of 4 weeks.

Shrew	Sample	Week 1	Week 2	Week 3	Week 4
#2	Saliva	38 ^a^	29,8 ^b^	27,85 ^c^	28,99 ^c^
	Lacrimal fluid	34 ^a^	nd*	27,3 ^c^	- ^c,^*
	Skin swab	36 ^a^	34,5 ^b^	29,48 ^c^	30,2 ^c^
	Urine	39 ^a^	- ^b^	30,07 ^c^	nd
	Faeces	- ^a^	36,3 ^b^	31,59 ^c^	- ^c^
	Lair	nd	nd	nd	32,96 ^c^
#5	Saliva	28,53 ^d^	30,36 ^d^	30,57 ^d^	32,50 ^d^
	Lacrimal fluid	32,15 ^d^	33,12 ^d^	34,48 ^d^	36,38 ^d^
	Skin swab	35,02 ^d^	34,91 ^d^	34,10 ^d^	36,06 ^d^
	Urine	34,68 ^d^	38,86 ^d^	34,83 ^d^	35,59 ^d^
	Faeces	- ^d^	- ^d^	- ^d^	- ^d^
	Lair	- ^d^	35,50 ^d^	- ^d^	38,92 ^d^
#6	Saliva	31,12 ^d^	33,31 ^d^	33,35 ^d^	31,27 ^d^
	Lacrimal fluid	35,06 ^d^	35,61 ^d^	36,60 ^d^	32,95 ^d^
	Skin swab	36,4 ^d^	36,97 ^d^	36,89 ^d^	32,97 ^d^
	Urine	37,34 ^d^	36,41 ^d^	- ^d^	34,47 ^d^
	Faeces	- ^d^	- ^d^	- ^d^	- ^d^
	Lair	35,64 ^d^	- ^d^	- ^d^	33,04 ^d^

Footnote Table 1: * BoDV-1 = Borna disease virus;— = negative; nd = not done

Results are presented as ct-values of different real time RT-PCR runs: a = first run; b = second run; c = third run; d = fourth run

For the investigation of long lasting virus shedding BoDV-1 infected animals were sampled again after at least more than 250 days in the husbandry. In these four naturally infected shrews(#2, #9, #10, #12), viral RNA was present in swabs from saliva, lacrimal fluid, skin and urine, but was not detectable in faeces (Table 2). Ct-values varied between different animals and between the samples but were lowest in saliva in two animals.

**Table 2:** Detection of BoDV-1* RNA in naturally infected bicolored white-toothed shrews after more than at least 250 days in husbandry.

Shrew	time point of sampling	Sample	ct-value
#2	634 days a.c. *	Saliva	32,44
		Lacrimal fluid	34,93
		Skin swab	nd*
		Urine	33,94
		Faeces	-*
#9	315 days a.c.	Saliva	27,4
		Lacrimal fluid	26,76
		Skin swab	31,64
		Urine	nd
		Faeces	-
#10	315 days a.c.	Saliva	25,95
		Lacrimal fluid	29,62
		Skin swab	33,93
		Urine	nd
		Faeces	-
#12	284 days a.c.	Saliva	34,47
		Lacrimal fluid	32,21
		Skin swab	33
		Urine	32,19
		Faeces	-

Footnote Table 2: * BoDV-1 = Borna disease virus; a.c. = after capture;— = negative; nd = not done; Results are presented as ct-values

**Fig 2:**
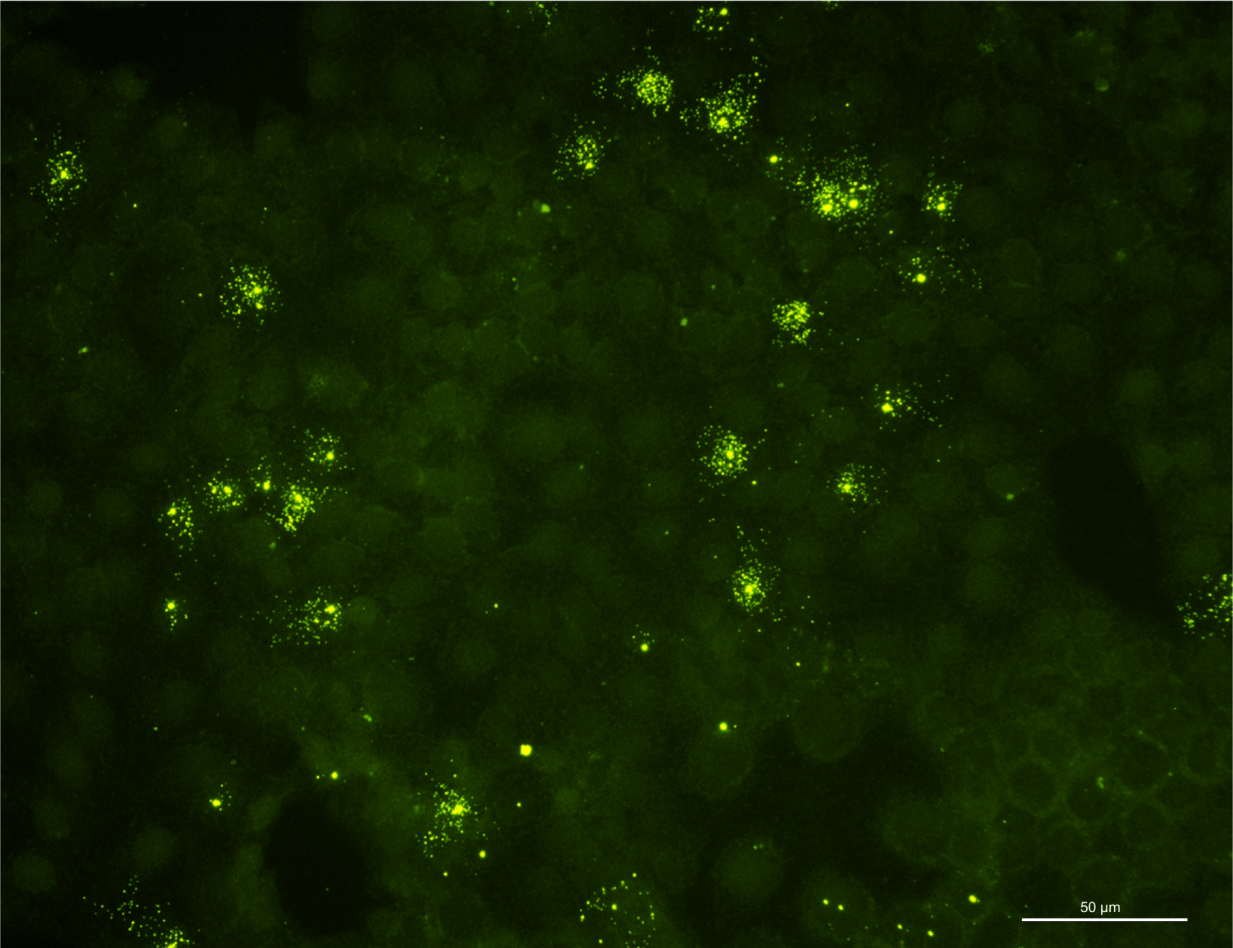
Detection of Borna disease virus-1 isolated from saliva (shrew #2) in rabbit embryonic brain cells (3^rd^ passage after isolation). Immunofluorescence, polyclonal anti-BoDV-1 rat serum.

**Fig 3:**
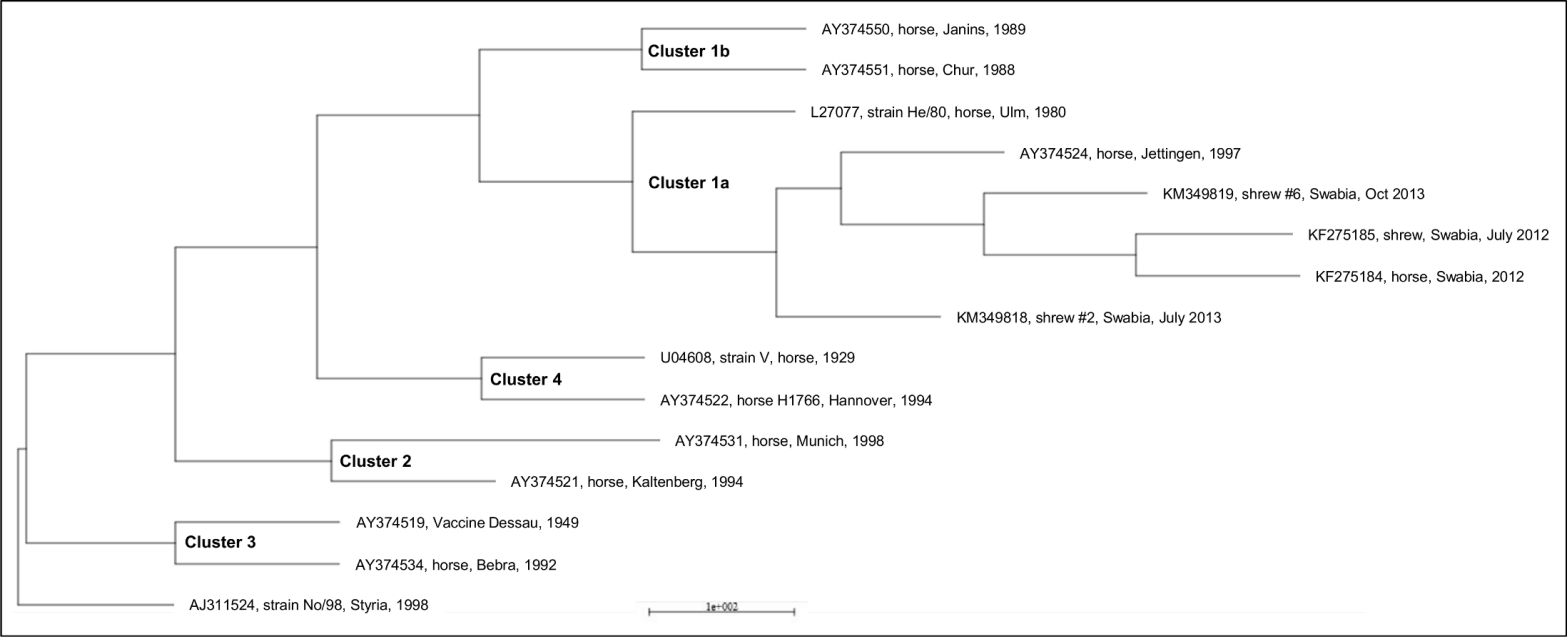
Phylogenetic analysis of BoDV-1 sequences obtained from isolated virus. 2150 nt long nucleic sequences comprising N, P, X genes of two isolated virus isolates (shrew #2, shrew #6), two sequences obtained from a shrew and a horse of the same region (KF275184, KF275185 [17]) and other representative BoDV-1 of endemic subclusters [25]. Cluster 1: Southwest Germany and Southern Rhine valley group with Cluster 1a: Baden-Wurttemberg and parts of Bavaria, Germany (L27077, AY374524) and Cluster 1b: Switzerland, The Principality of Liechtenstein and Austria (AY374550, AY374551), Cluster 2: South German Group (AY374521, AY374531), Cluster 3: Southern Saxony-Anhalt and Saxony (AY374519, AY374534), Cluster 4: Central German group (U04608, AY374522). Tree is rooted with BoDV-2 (AJ311524).

Furthermore, infectious virus was successfully isolated on REB cells from all of the BoDV-1-positive shrews caught in 2013 (#2, #5, #6) in samples from saliva (#2, #6), skin/sebum (#2, #6) and urine (#5, #6) (Fig 2). Viral RNA of isolates from saliva of #2 and saliva of #6 was sequenced (Genbank accession no. KM349818, KM 349819). In a 2150 nucleotide stretch (nt 17 to 2161 covering the N, X, P, half of M-protein-encoding regions) sequences of both isolates revealed a homology of 99% compared to a recent BoDV-1-sequence (GenBank accession no. KF275185) obtained from a shrew of the same endemic area and to an equine BoDV-1-sequence from a horse housed in the same region (GenBank accession no. KF275184)[17]. Both isolates are part of the regional BoDV-1 subcluster 1a. (Fig 3)

**Fig 4:**
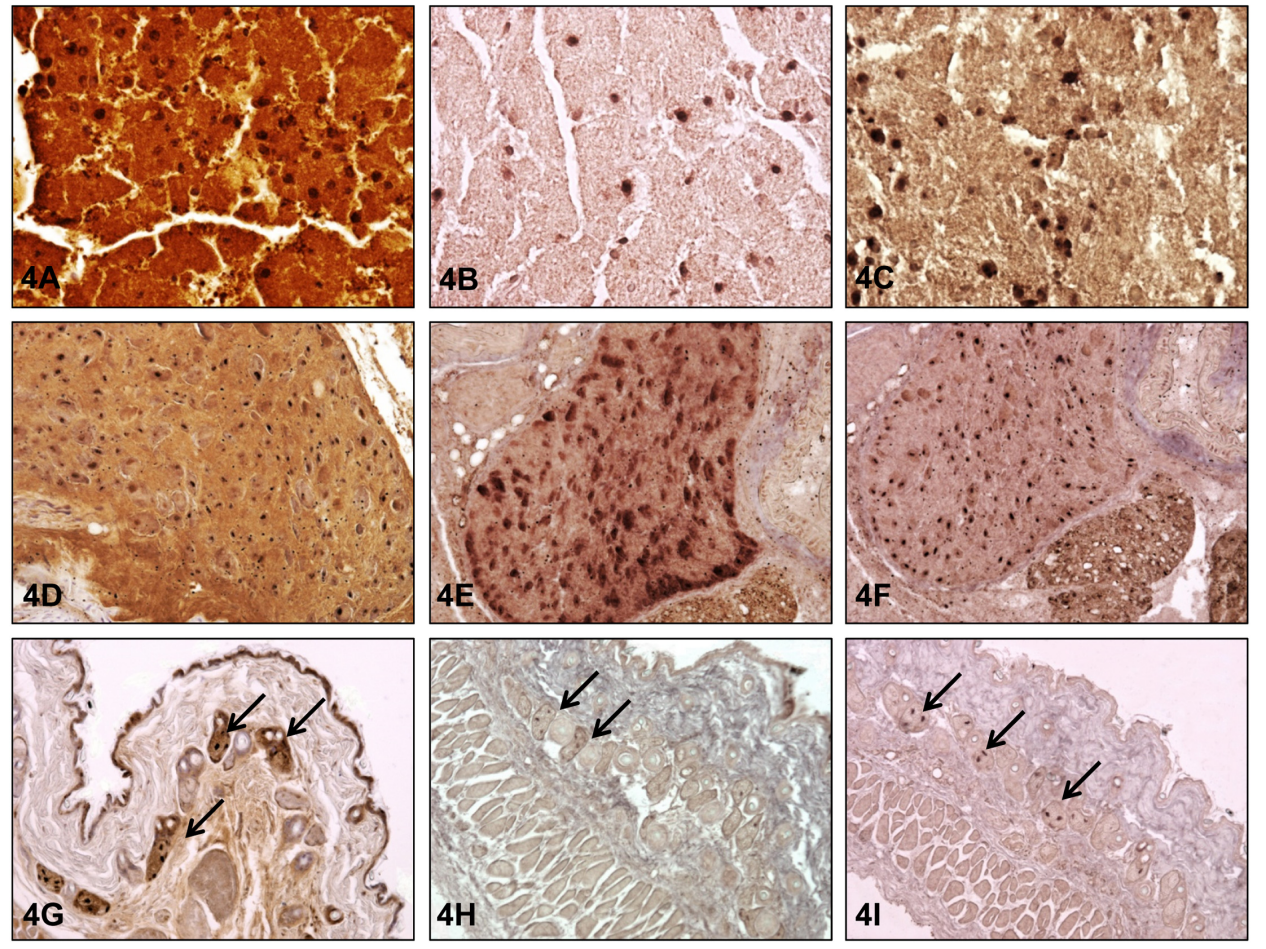
Demonstration of BoDV-1 nucleoprotein, messenger RNA and genomic RNA. (A) Demonstration of BoDV-1 nucleoprotein by immunohistochemistry (IHC) in the brain of *C*. *leucodon* #5017; (B) Demonstration of BoDV-1 messenger RNA by in-situ hybridization (ISH) in the brain of *C*. *leucodon* #5017; (C) Demonstration of genomic BoDV-1 RNA by ISH in the brain of *C*. *leucodon* #5017; (D) Demonstration of BoDV-1 nucleoprotein by IHC in the trigeminal ganglion of *C*. *leucodon* #2001; (E) Demonstration of BoDV-1 messenger RNA by in-situ hybridization (ISH) in the trigeminal ganglion of *C*. *leucodon* #2001; (F) Demonstration of genomic BoDV-1 RNA by ISH in the trigeminal ganglion of *C*. *leucodon* #2001; (G) Demonstration of BoDV-1 nucleoprotein by IHC in the skin, mainly in the sebaceous glands of *C*. *leucodon* #2001; (H) Demonstration of BoDV-1 messenger RNA by in-situ hybridization (ISH) in the skin, mainly in the sebaceous glands of *C*. *leucodon* #2001; (I) Demonstration of genomic BoDV-1 RNA by ISH in the skin, mainly in the sebaceous glands of *C*. *leucodon* #2001;

The results obtained from the living shrews were compared to the organ distribution of viral antigen and BoDV-1-RNA in three naturally BoDV-1-infected *C*. *leucodon* from pest control (#2001 and #5017, [17] and another animal #5072 from the same stable as #5017) and in the two deceased shrews. Detailed information about organ distribution is given in S3 Fig. In all of these animals, mRNA, genomic RNA and/or viral antigen were detected in the nervous system and widespread in peripheral organs (e.g. epithelial cells of the parotid gland, lacrimal gland, sebaceous glands, bronchi, kidney tubules, esophagus and epidermal keratocytes) [17] (Fig 4). Interestingly, viral antigen was also present in the uterus in one shrew. Thus, detection of viral RNA and infectious virus from secretions and excretions in the living shrews (saliva, lacrimal fluid, skin swabs, urine and faeces) fits well with the morphological demonstration of viral antigen and RNA in the respective organ systems and further confirms successful viral replication in peripheral organs. Beside virus shedding via secretions and excretions shedding of BoDV-1 seems also to be possible via scaling of epidermal epithelial cells.

## Discussion

Reservoir-bound RNA viruses reside typically inconspicuously in animal reservoirs such as bats, rodents and insectivores. However, transmission routes, host-pathogen interactions necessary for viral maintenance in the respective animal population and factors needed to cross the species barrier are still rudimentarily known. Thus, reliable animal models are urgently needed. The order *Mononegavirales* comprises many viruses with high zoonotic and pathogenic properties, e.g. *filoviruses*, *henipaviruses*, *paramyxoviruses* and *lyssaviruses* which reside in bat reservoirs [7, 31]. In their biological behaviour, bornaviruses, as known from the mammalian Borna disease virus-1 (BoDV-1), are unique [10, 32], but in several aspects pretty comparable to other neurotropic *Mononegavirales*. The recently found zoonotic variegated squirrel 1 Bornavirus (VSBV-1) clearly differs in its homology to the classical mammalian BoDV-1 but provides evidence for its zoonotic capacities [10]. As the current knowledge is sparse, it is not known if VSBV-1 share features with BoDV-1 behaviour. However, detection of VSBV-1 in several organs including CNS and peripheral organs like lung and kidney of the squirrel [10] also indicate a widespread virus distribution comparable to the BoDV-1 infected bicolored white-toothed shrew.

Typically shrews rear up to four litters from March to September and winter resource shortage is the most important source for mortality [33]. Trapping took place during summer and autumn, therefore caught shrews were likely born in the same year and the age at time of trapping could be estimated between 1 to 6 months. As most of the offspring settles locally [33] kinship between the individuals and joint rearing cannot be excluded.

During trapping, infection status of the individuals was unknown. Previous studies showed different infection prevalence of shrews that also differed between the trapping sites in the study. Hilbe et al [14] found only infected shrews (100%), Puorger et al. [15] detected 2/6 infected shrews (33%), Bourg et al. [17] showed 1/1 infected shrews (100%) at one site und 1/19 infected shrews (5%) at the other site whereas Dürrwald et al. [18] found an amount of 9/17 infected shrews (53%) at one site with a variance between the years from 25% to 100%. These differences can be due to the small number of animals in the respective population or represent the natural variation within the shrew population between sites and years. Since examination of larger cohorts has not been carried out so far, the percentage of naturally infected shrews among the trapped animals could not be predicted in detail. Six naturally infected shrews out of eleven shrews implies a percentage of 55% of infected shrews with variations between the sites and years from 50% (site A, year 2013 3/6, year 2014 1/2) to 66% (site B, year 2014 2/3). As all non-infected animals did not show any shedding during the whole observation period, transmission of the virus in the husbandry could be successfully prevented in captivity.

Current data from living shrews provide reliable evidence that natural BoDV-1-infection in these animals is indeed clinically inconspicuous over a long time period as already previously assumed [15, 18] despite persistent infection with shedding of infectious virus via various sites. During the observation period of up to 600 days, only two naturally infected animals were lost due to an intestinal invagination in one case and hepatitis/pneumonia in the other case which did not seem to be directly related to BoDV-1 infection. In the bronchial epithelium of the animal suffering from hepatitis/pneumonia only few cells harboured BoDV-1 nucleoprotein, BoDV-1 mRNA and genomic RNA without associated distribution to the pneumonia and in the liver only genomic RNA was detected in very few cells.

Interestingly, shedding of viral RNA was continuously present.As shrews were naturally infected before trapping, the time between the infection and first virus release remain unknown. However, low ct-values were found in samples taken at time points at least more than 4 to 8 weeks after infection and at time points at least more than 200 days after infection. This indicates a persistent BoDV-1 infection as known from other animals [11, 13] with long lasting and continuous virus release. There was certain variability in the amount of viral RNA, sites of shedding, between individual animals and for the time points of sampling. Some of these variations can be due to variations in sample size, as gathering of samples had to be performed non-invasive on non-anaesthesized animals. However, several shrews exhibited lowest ct-values in saliva and lacrimal fluid regardless of time point of sampling. Whether this might have a role for virus transmission, e.g. combating, needs to be further investigated.

The simultaneous presence of viral antigen, viral mRNA and genomic RNA in CNS and peripheral tissues points to many sites of viral replication thereby enhancing probability of successful virus transmission to other animals [17]. Horizontal transmission of BoDV-1 in shrews might be either achieved via direct contact with secretions or excretions or even via contaminated environment. Since shrews are known to behave territorially, infection by infected saliva during combating for a habitat might also occur. Vertical transmission of BoDV-1 in shrews cannot be excluded as viral antigen has been detected in the uterus. However, the route of entry in the reservoir still remains unknown. Offspring might already be infected early by their mothers due to the various sites of viral shedding even from the skin. The underlying viral mechanisms of maintenance in the reservoir are still incompletely understood but might include adjusted viral life cycle possibly with attenuated pathogenicity, differences in viral entry and circumvention of the antiviral host immune system [4, 34, 35]. The latter could be achieved best in specific situations of the host immune system. Infection of animals in an immune-incompetent stage can lead to persistent, immune-tolerant virus infections, often associated with shedding of high doses of infectious virus and without any severe clinical signs and notable inflammatory lesions. To date it remains unknown whether disseminated BoDV-1 infection of shrews is only possible when infected in an immune incompetent state as known for rats [19]. However, the clinical inconspicuous course could point to an immune tolerant infection and a highly adapted viral-host interaction. Neonatally BoDV-1 infected rats display no neurological signs but increased motor activity, learning deficits and subtle changes in social behaviour and memory [36, 37]. Moreover, experimental BoDV-1 infection of the prosimian tree shrew (*Tupaia glis*) leads to a persistent infection and transient mild encephalitis, resulting in a disorder characterized primarily by hyperactivity and pronounced disturbances in social and breeding behavior rather than neurological signs [38]. In the neonatally BoDV-1 infected rat the behavioral changes were attributed to lesions in the hippocampus and cerebellum and in the tree shrew to alterations of the limbic system. Whether naturally BoDV-1 infected shrews also display subtle deficits in learning, memory and/or social behavior, especially mating, needs to be addressed in further behavioral and breeding experiments. As known so far, *C*. *leucodon* did not exhibit any morphological changes in cerebellum, hippocampus or elsewhere in the brain as noted for the neonatally infected rat. However, any behavioral changes might contribute to higher contact frequency or increased aggressive and territorial behavior thereby facilitating viral transmission and maintenance in the reservoir.

Characteristics of the shrew population correspond well to the epidemiologic pattern of Borna disease. The distribution of *C*. *leucodon* in Bavaria and the prevalence of Borna disease seem to be connected [16]. The yearly varying peaks of Borna disease in accidental hosts and the decline of Borna disease within the last decades could be related to population dynamics of the shrews between the years and the restriction of habitats indirectly caused by modern agriculture [18]. Inbreeding and low dispersal distance of the offspring correlates with the limited distribution of BoDV-1 within endemic territories [18].

Moreover, the continuous secretion and excretion of infectious BoDV-1 and the detection of viral RNA in the lair substantiates the hypothesis that “infectious dust” is responsible for BoDV-1 transmission to accidental hosts through the intranasal route as known for hantavirus infections [39]. In this scenario, the BoDV-1 infection of horses and sheep might rather represent an accidental occasion. To date it still remains to be solved whether and which factors are responsible for successful crossing the species barrier. Amount of infectious virus, virulence, immune status and age of reservoir and accidental host as well as their genetic makeup might function as essential co-factors.

Taken together, shedding of BoDV-1 in the bicolored white-toothed shrew is achieved via various routes which enable successful viral maintenance in the reservoir population and even fatal transmission to susceptible accidental hosts such as horses and sheep. Moreover, these animals serve as suitable model to investigate host and pathogen factors that enable persistent viral co-existence in apparently healthy carriers.

## Supporting Information

S1 Fig Body mass change infected individuals [g]. (TIF)

S2 Fig Body mass change non-infected animals [g]. (TIF)

S3 Fig Organ distribution of BoDV-1 nucleoprotein, mRNA and genomic BoDV-1 RNA of individual shrews. (PDF)

S4 Fig No detection of BoDV-1 antigen or RNA in a non infected shrew by immunohistochemy and in-situ hybridization, shrew was trapped dead by pest control. (TIF)

S1 Table Overview of individual shrews of the study. (DOCX)
