# Supplementary figures and images for: Shedding of Infectious Borna Disease Virus-1 in Living Bicolored White-Toothed Shrews
Source: PLoS One. 2015 Aug 27;10(8):e0137018. doi: 10.1371/journal.pone.0137018 (PMC4552160; doi:10.1371/journal.pone.0137018)

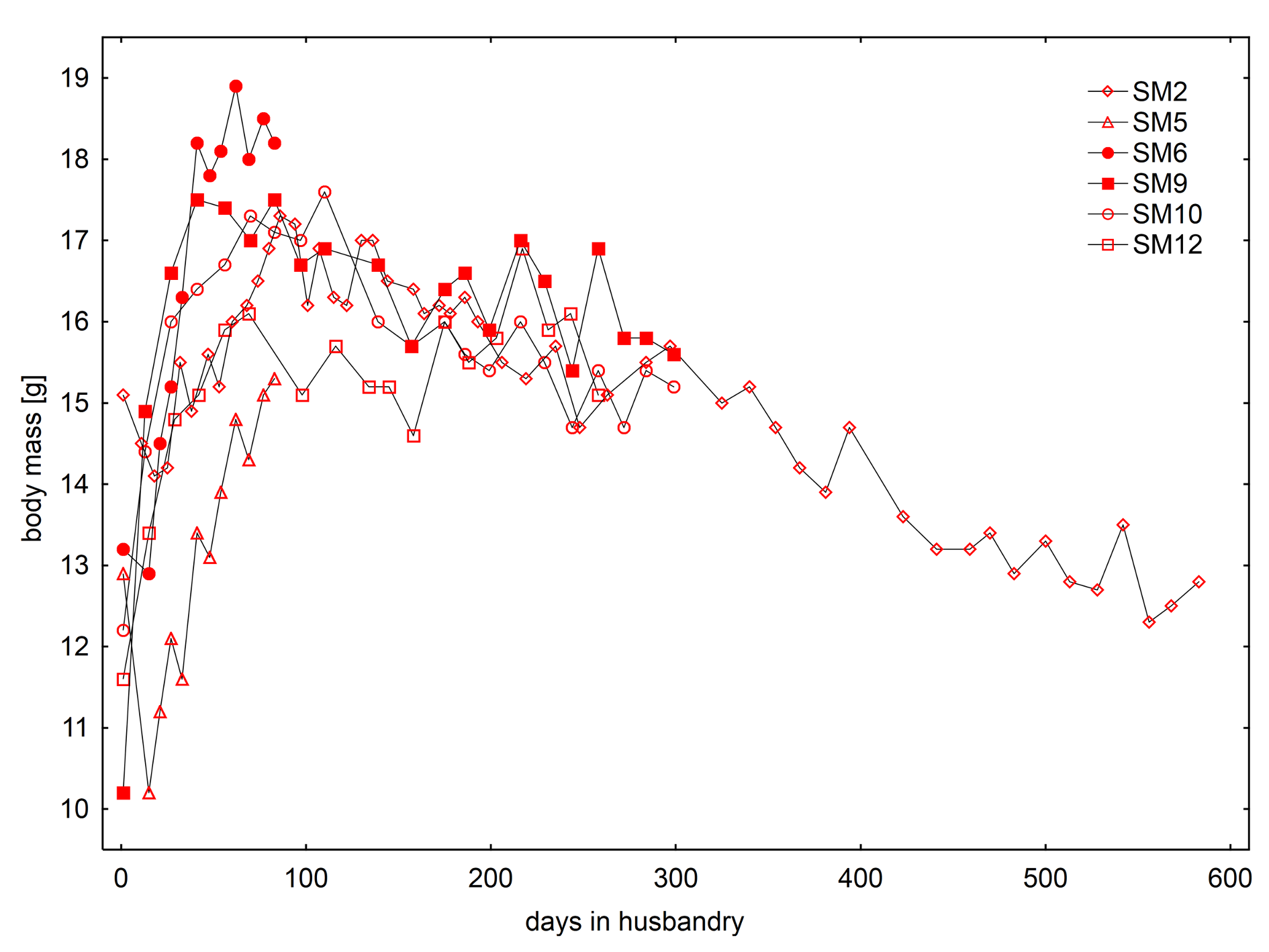

Supplement: S1 Fig — (TIF) [file pone.0137018.s001.tif]

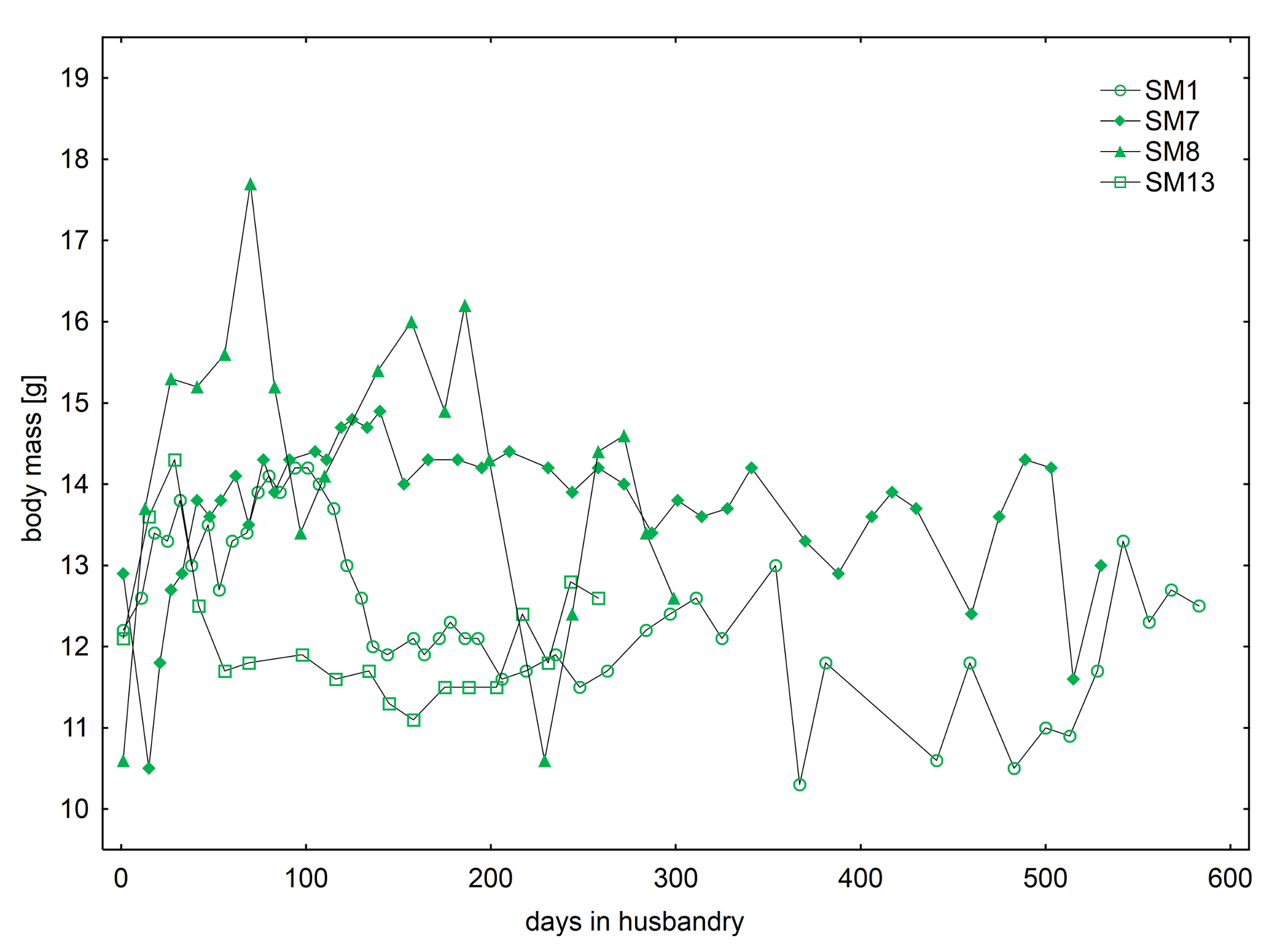

Supplement: S2 Fig — (TIF) [file pone.0137018.s002.tif]

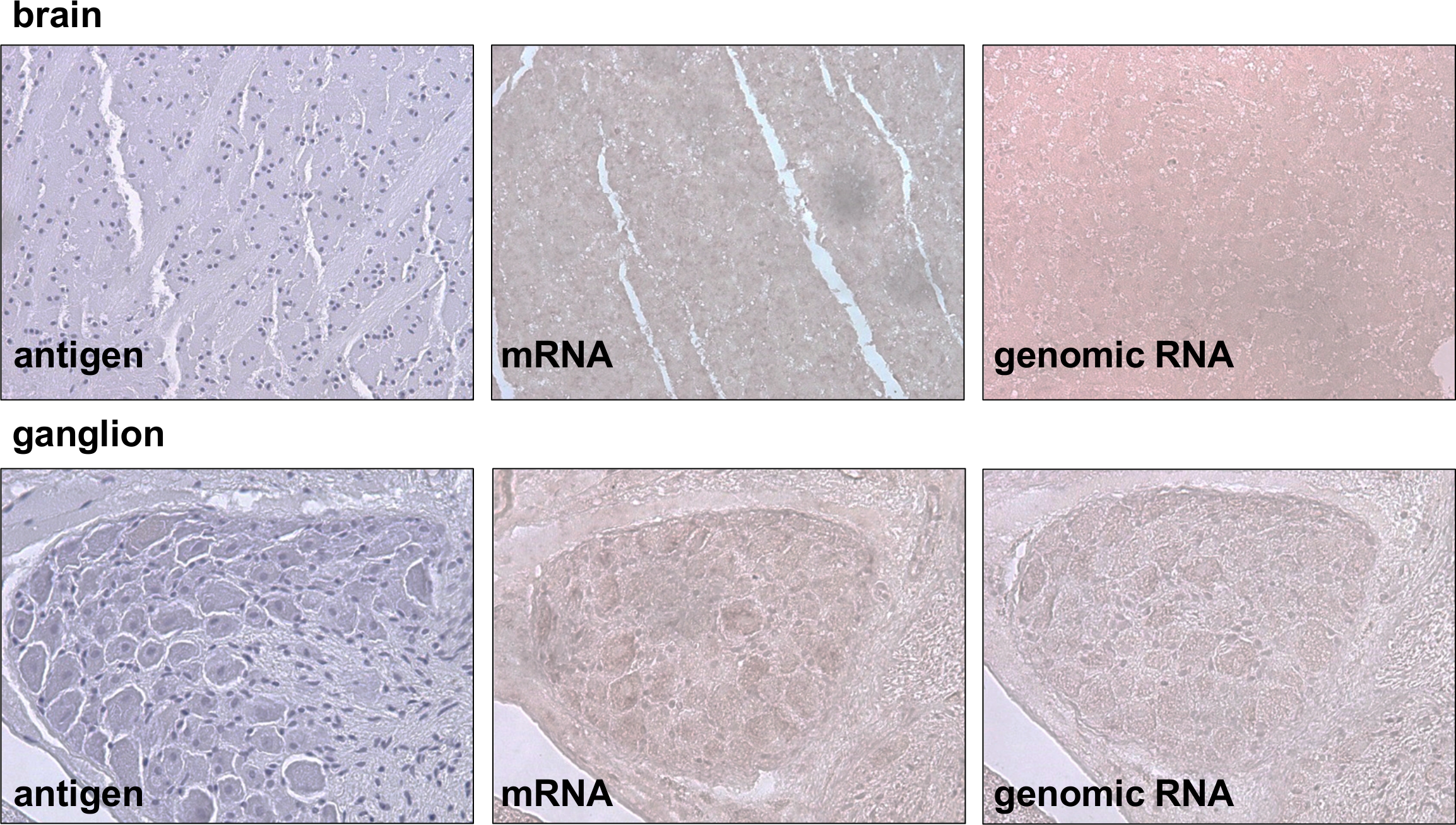

Supplement: S4 Fig — (TIF) [file pone.0137018.s004.tif]
