# Supplementary material for: Shedding of Infectious Borna Disease Virus-1 in Living Bicolored White-Toothed Shrews
Source: PLoS One. 2015 Aug 27;10(8):e0137018. doi: 10.1371/journal.pone.0137018 (PMC4552160; doi:10.1371/journal.pone.0137018)
Supplement: S3 Fig — (PDF) [file pone.0137018.s003.pdf]

Overview of detection of BoDV-1 nucleoprotein, mRNA and genomic RNA in BoDV-1 infected bicolored white-toothed shrews

	#2001			#5017			#5072		
	male			female			female		
	IHC	ISH gen. RNA	ISH mRNA	IHC	ISH gen. RNA	ISH mRNA	IHC	ISH gen. RNA	ISH mRNA
Brain	+++	+++	+++	+++	+++	+++	+++	+++	+++
Trigeminal ganglion	+++	+++	+++	+	+	+	++	+++	+
Peripheral nerves	+++	+++	+++	+	+	+	+++	+(+)	+
Salivary gland	(+)	++	++	+	-	-	+	(+)	(+)
Lacrimal gland	+(+)	++	++	not evaluable	not evaluable	not evaluable	+(+)	+++	++
Heart	not evaluable	not evaluable	not evaluable	not evaluable	not evaluable	not evaluable	-	-	-
Lung, bronchial epithelium	-	-	-	+	-	-	+	++	+
Esophagus	++	+	++	not evaluable	+	+	-	++	++
Liver	-	-	-	-	-	-	-	-	-
Spleen	not evaluable	not evaluable	not evaluable	not evaluable	not evaluable	not evaluable	not evaluable	not evaluable	not evaluable
Intestine, muscular layer	-	-	-	-	-	-	-	-	-
Kidney, tubulus epithelium		not evaluable	not evaluable	+	+	+	-	-	-
Skin, corneal epithelium	+	+	+	+	+	+	-	k.A	k.A
Sebaceous gland	+++	+++	+++	+++	+(+)	++	not evaluable	-	-
Olfactory epithelium	++	+	+	+	+	+	++	+++	++(+)
Spinal cord	+++	+	+	+++	+++	+++	+++	+++	++
Spinal ganglion	(+)	not evaluable	not evaluable	+	+	+	++	+++	+++
Mamma	not evaluable	not evaluable	not evaluable	not evaluable	not evaluable	not evaluable	-	-	-
Uterus	not evaluable	not evaluable	not evaluable	not evaluable	not evaluable	not evaluable	++	++	+
Testis	+(+)	(+)	+	not evaluable	not evaluable	not evaluable	not evaluable	not evaluable	not evaluable

+++ = high signal; ++ = moderate signal; + = low signal; - = no signal
IHC = immunohistochemistry; ISH = in-situ hybridization

	#5			#6		
	male			female		
	IHC	ISH gen. RNA	ISH mRNA	IHC	ISH gen. RNA	ISH mRNA
Brain	++	+++	++	+++	++	+
Trigeminal ganglion	++	++	++	++	++	++
Peripheral nerves	++	+	+	++	++	+
Salivary gland	++	++	+	+	++	++
Lacrimal gland	++	++	-	++	++	+
Heart	-	-	-	-	-	-
Lung, bronchial epithelium	++	++	++	+	(+)	(+)
Esophagus	++	+	-	+	-	-
Liver	-	-	-	-	+	-
Spleen	+	-	-	-	-	-
Intestine, muscular layer	+	++	-	++	++	+
Kidney, tubulus epithelium	-	+	-	+	(+)	(+)
Skin, corneal epithelium	-	+	-	++	++	+
Sebaceous gland	-	+	-	++	++	+
Olfactory epithelium	+	++	-	+	+	-
Spinal cord	++	+	+	++	++	++
Spinal ganglion	++	+	+	++	++	++
Mamma	not evaluable	not evaluable	not evaluable	-	-	-
Uterus	not evaluable	not evaluable	not evaluable	not evaluable	not evaluable	not evaluable
Testis	not evaluable	not evaluable	not evaluable	not evaluable	not evaluable	not evaluable

+++ = high signal; ++ = moderate signal; + = low signal; - = no signal
IHC = immunohistochemistry; ISH = in-situ hybridization
