# Supplementary material for: Shedding of Infectious Borna Disease Virus-1 in Living Bicolored White-Toothed Shrews
Source: PLoS One. 2015 Aug 27;10(8):e0137018. doi: 10.1371/journal.pone.0137018 (PMC4552160; doi:10.1371/journal.pone.0137018)
Supplement: S1 Table — (DOCX) [file pone.0137018.s005.docx]

| shrew | sex | time of trapping | trapping site | days in husbandry | BoDV-1 infected | trial group |
| --- | --- | --- | --- | --- | --- | --- |
| #1 | male | July 2013 | site A | > 600 | - |  |
| #2 | female | July 2013 | site A | > 600 | + | 1^*^, 2^*^ |
| #3 | female | September 2013 | site A | > 500 | - |  |
| #5 | male | October 2013 | site A | 70 | + | 1 |
| #6 | female | October 2013 | site A | 70 | + | 1 |
| #7 | male | October 2013 | site A | > 500 | - |  |
| #8 | female | June 2014 | site B | > 300 | - |  |
| #9 | male | June 2014 | site B | > 300 | + | 2 |
| #10 | male | June 2014 | site B | > 300 | + | 2 |
| #12 | male | July 2014 | site A | > 250 | + | 2 |
| #13 | male | July 2014 | site A | > 250 | - |  |

Footnotes Table S1: * trial group 1 = shrews that received intensive initial health monitoring; trial group 2 = shrews sampled for investigation of long lasting virus shedding
